# Population health management fit lifecycles in analytics

**DOI:** 10.3389/frai.2025.1496945

**Published:** 2026-04-09

**Authors:** James Andrew Henry

**Affiliations:** Institute of Biomedical Sciences, London, United Kingdom

**Keywords:** AI digital regulation service, population health management, human phenotype ontology, biological modeling, classify predictors and intercepts

## Abstract

**Introduction:**

“Population Health Management (PHM), Fit Lifecycles in Analytics” examines the policy and practice of AI-driven methodologies to enhance public health and patient safety in the context of the Human Phenotype Ontology (HPO). It aims for personalized healthcare delivery through the risk stratification of predictors and pathology segmentation for intercepts. This manuscript aimed to introduce the Five-Point PHM strategy as a mission for public trust and governance. Scientific and technological advancements address public genomic inclusiveness and engage biobanks and life sciences for national public health and patient safety oversight.

**Methods:**

The study assesses genome and socio-environmental health factor variables that segment and image disease through stakeholder engagement in real-world settings such as HPO neighborhood trials. It assesses practices for data training, emphasizing data alliances, scientific themes, and data structure. The manuscript evaluates data preprocessing and prioritizes open-source frameworks to ensure data balance and bias mitigation.

**Actions:**

The recommendation for a PHM infrastructure ensures personal classification under the national authority with a comparative analysis of AI architectures that highlights trade-offs in AI modes for HPO. Structural and continuous control monitoring, explainability, and model performance metrics are emphasized. The actions transform the vision and language for HPO, advocating for a national generative classification for genome predictor pre-eXam and eXam intercepts. Actions on guardrails and ethics address a secure and safe national program.

**Discussion:**

The governance of fit lifecycles in analytics discusses improvement with research science integration and accountability for HPO as primary care. The PHM mission and ten-year infrastructure plan addresses implementation challenges through government principles for an adoption mission with AISI/AIDRS authority. Diligent PHM through HPO policy action GPT-5, with federated data learning and quantum computing as emerging technologies that synergize for BM of predictors and intercepts.

**Conclusion:**

The manuscript concludes with the potential of the proposed PHM mission to support the UK AI Action Plan and principles outlined in the UK Government AI Playbook. By integrating research science into HPO evidence-based primary care practice, this paper drives progress in public health and patient safety for national well-being and growth. The study advocates for the ethical and secure implementation of AI-driven PHM with public science and technology trustworthiness.

## 1 Introduction to population health management and fit lifecycles in analytics

The Population Health Management (PHM) mission incorporates Human Phenotype Ontology (HPO) policy and ensures actionable and sustainable analytics for the NHS primary care of Biological Modeling (BM) (NHS England, 2023; Putman et al., 2023). Fit lifecycles in analytics serve as an action plan for the Department of Science, Innovation, and Technology (DIST), aiming to develop a real-world AI infrastructure for humanity, as nations digitize citizen records from multimodal scientific data themes (Department of Science, Innovation and Technology, 2023; United Nations, 2024). The Genomics England network users and Life Science division steward PHM, with valid multi-omics and social-environmental data analytics across biobanks (Genomics England, 2024a; GOV.UK and Industrial Strategy, 2025; UK Biobank, 2024b). The strategy envisions nations engaged in a UK-wide AI Digital Regulation Service (AIDRS) with safe and secure Quantum Agentic AI for personalized well-being (Welsh Government, 2024).

**Figure 1:**
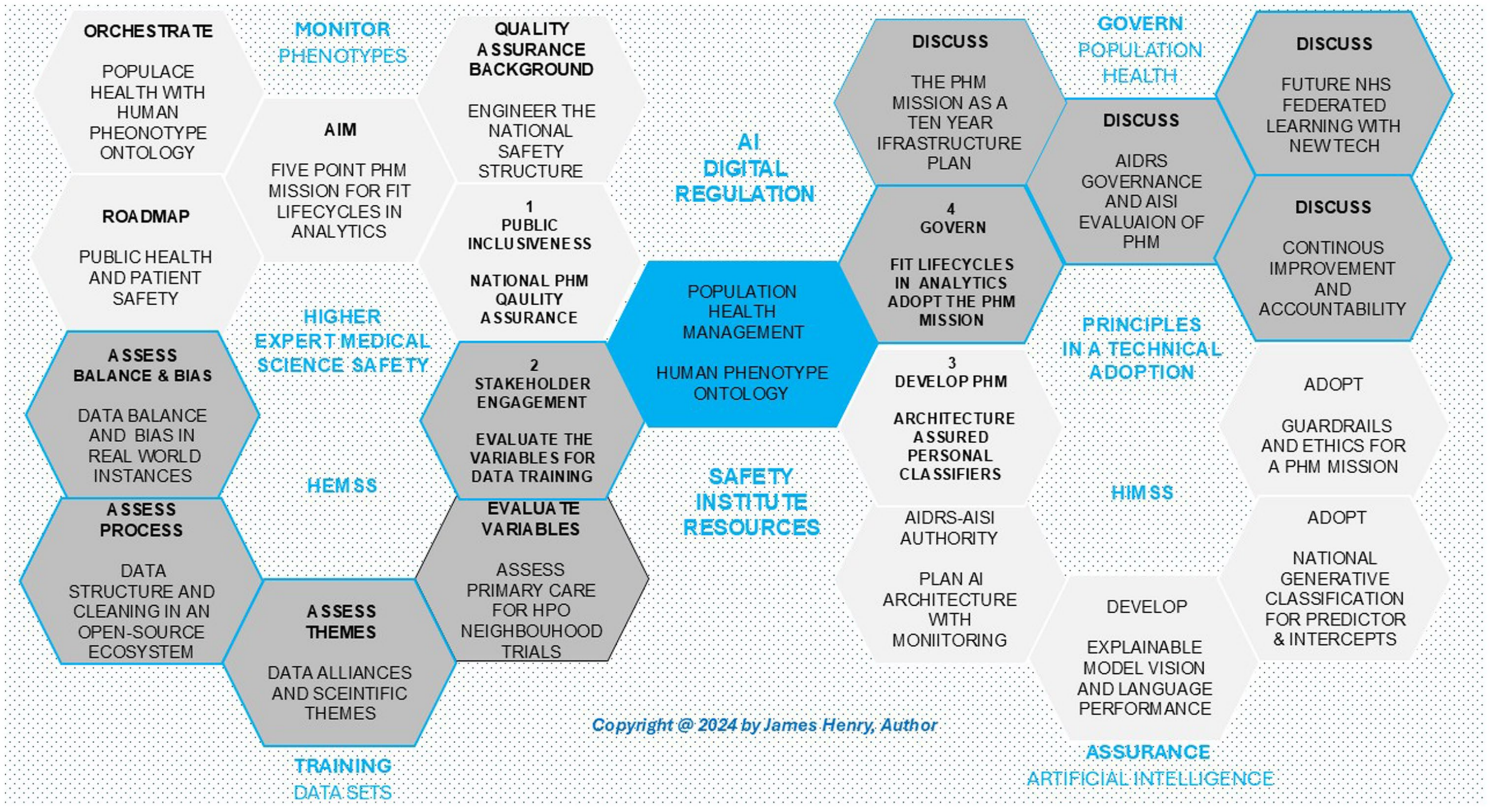
Population health management the roadmap for human phenotype ontology adoption.

In Figure 1, the author proposes the PHM mission in a roadmap for HPO adoption of BM as primary care, aligning global scientific research with international technology infrastructure for public trustworthiness in classifying predictors and intercepts in each citizen lifecycle. Figure 1 provides an overview of the manuscript for fit lifecycles in future analytics, illustrating national quality aims, data training, AI analytics assurances, and stewardship for public health and patient safety as science and technology stakeholders build a conventional medicine ecosystem for continuous improvement in clinical support decisions.

## 2 Aims for national PHM quality assurance with public inclusiveness

UK national strategic aims for the PHM of HPO require quality assurance (QA) in current research studies for routine projects to align a generation of newborn genomic screens with our future phenotypic health, with full public inclusiveness in what is fair and just (Genomics England, 2024b; Our Future Health and NHS, 2022). Therefore, the manuscript goals for national QA, which centralize people in the plan, include Section 2.1, “The QA Background to Engineer the National Safety Infrastructure;” Section 2.2, “The Five-Point PHM Mission for Fit Lifecycles in Analytics;” Section 2.3, “Orchestrate Population Health with the Human Phenotype Ontology,” and Section 2.4, “Roadmap for Public Health and Patient Safety.”

### 2.1 The QA background to engineer the national safety infrastructure

National QA in conventional medicine and traditional diagnostics has been mapped over the previous decade to project science and technology for personalized healthcare in an NHS 10-Year plan, engineering analytics in life cycles as an unambiguous safe space (Department of Health and Social Care, 2025). The QA background reports and reviews include:

- The Francis Report highlighted the need for a patient-centered transformation across healthcare services, emphasizing transparency and accountability following a series of unfair and unjust well-being incidents (Francis, 2013). Indeed, NHSE published a Pathology QA Review to improve pathology services, which set the stage for PHM (Pathology Quality Assurance Review, 2014).
- The Topol Review prepared the healthcare workforce to deliver the digital future, setting a vision to lead globally in technology, analytics, genomics, digital medicine, and AI (Topol, 2019). The Royal College of Pathologists detailed the pre-cycle to integrate scientific themes into national governance (Get It Right First Time, 2021).
- The Goldacre Review recommended better and broader use of health data for research analysis, aiming for the safety and security of health data (GOV.UK, 2022). The RCPath endorsed standard variations and published strategic guidance for point-of-need testing that could digitize and deploy HPO analytics (Royal College of Pathologists, 2022; Institute of Biomedical Sciences, 2023).
- The Hewitt Review of Integrated Care Systems called for improved governance with continual improvement and enhanced accountability for preventative services and interventions (GOV.UK, 2023a). The UK AIDRS was developed for the PHM ecosystem in a future of HPO predictors and target therapy in need of transparent governance (NHS, 2024).
- The Darzi Report provided an analysis of the NHS gaps, enhancing engagement in preventative healthcare plans with advanced infrastructure technologies (Darzi, 2024). The UK-US Memorandum of Understanding on AI was subsequently developed within international safety/security institutes, serving as a global health foundation in the coming decade (UK.GOV, 2024).

The author’s initial QA program proposal, developed under the supervision of NHSE, laid the groundwork for an end-to-end workflow that now supports national PHM QA proposals. That framework was closely aligned with ISO 15189:2022 Annex A to govern, train, and assure truth in point-of-need arrangements, which advance in global intelligence ecosystems (Henry, 2014).

### 2.2 The five-point PHM mission for fit lifecycles in analytics

The five-point PHM mission for fit lifecycles supports the NHS’s 10-year plan for genomics and life sciences with capacity for public health risk stratification and intelligent pathology capabilities that target therapy in “promising” analytics (Department of Health and Social Care, 2025; Genomics England, 2024a; GOV.UK and Industrial Strategy, 2025; Oddy et al., 2024). The NHS is establishing a PHM infrastructure for our HPO identity as agile groups develop HIMSS maturity (Healthcare Information and Management Systems Society, 2019). Meanwhile, the mission for fit lifecycles in analytics to predict and intervene in pathology involves stewarding government plans when actioned with Higher Expert Medical Science Safety (HEMSS), as proposed by the author and depicted in Figure 1.

#### 2.2.1 HIMSS maturity for agile method development of HPO projects

Figure 1 illustrates the road map for HPO adoption within PHM, demonstrating the alignment of HIMSS maturity with Government Digital Services and agile development for PHM learning. This ecosystem, detailed across Sections 2–4 of the figure and Sections 3–5 of the text, requires robust data training with AI assurances while stewarding the future of fair HPO. A five-point PHM mission with HPO policy as primary care is a digital landscape of national initiatives that include the following points:

1. Genomics iterative development gains insights into pathology predispositions with tools for public health research and practice, as variants facilitate personalized HPO treatment plans, which need expansion in 2026 (Department of Health and Social Care, 2022).
2. Imaging testing visualizes HPO for pathology diagnosis and changes after therapy with equitable access to detect early signs of disease, monitor progression, and evaluate treatment efficacy (England, 2025a).
3. Social determinants of health address socioeconomic status, education, and access to healthcare, which influence public health outcomes as we address health equity with community interventions (Public Health England, 2017).
4. Biobank research visions the predictors and intercepts in a PHM ecosystem to access samples for real-world HPO instances that scan data in points 1–3 for BM (GOV.UK, 2021a).
5. Life Science data collaborative efforts in national integration enhance the ability to predict and intercept BM/HPO in disease, leading to more effective prevention and treatment strategies (GOV.UK, 2024c).

#### 2.2.2 HEMSS principles for the adoption of HPO projects

HEMSS accelerates the PHM mission for seamless HPO project adoption with policy for ecosystem improvement as the primary care, supported by standards, tools, and classifications (Henry, 2025b; Henry, 2025c). HEMSS operates with AIDRS stewards or across authorities; if chosen for a national or global health strategy, it will oversee safe and secure Agentic AI (Topol, 2019; Henry, 2025a). HEMSS principles prioritize public inclusiveness in national QA initiatives while engaging stakeholders in data training and AI assurance, as classified by Gen AI experts for genomics truth in value-based point-of-need adoptions (Department of Health and Social Care, 2022; Institute of Biomedical Sciences, 2023). A description of HEMSS stewardship sustains multidisciplinary cooperation and proposes the following:

- Higher infrastructure, classification, and stewardship align hybrid servers on platforms for statistical-based predictive and diagnostic analytics with precise care capabilities in scalable HPO management, ensuring fluid integration and real-time data processing for clinical support decisions.
- Expert agile BM development, which classifies the WGS pre-eXam pathology and extends an autonomous, precise care eXam intervention with probable or evidence-based insight from an AI infrastructure that facilitates HPO personalization in a PHM ecosystem.
- Medical practitioners and biopharma stakeholders engage in classifications for truth in biological and socioenvironmental scientific data at the point of need across Primary Care Networks, cooperating cohesively to create personalized workflows in health and social care.
- Science data in multi-omics and social factor themes involve the public in developing digital twin classifications from their multimodal data, which in turn accumulate in upstream informatics for analytics across a PHM ecosystem, which adopts HPO as value-based care.
- Safety for patients, public health, and parity evolve in a PHM mission for HPO primary care, ensuring consistent tools and standards for uninterrupted BM and care delivery in both the diagnosis and personalized interventions within mental and physical disorders.

### 2.3 Orchestrate population health with the human phenotype ontology

The DSIT improves upon national NHSE goals in how to orchestrate PHM services for HPO determinants that predict health and precision value-based care (NHS England, 2023; Putman et al., 2023; Department of Science, Innovation and Technology, 2023). On the horizon, government and NHS providers orchestrate the PHM of HPO in a five-point mission as science and technology adopt AI transformers to meet public needs (GOV.UK, 2024e; van Ede et al., 2023). The AI Safety/Security Institute’s role in BM pre-testing is instrumental, albeit in its infancy, for the risk stratification to segment pathology predictors and intercept conditions (NIST, 2024).

Therefore, HEMSS stewarding principles build on HIMSS to develop scientific data themes and adopt transformer technology to inform the public about rare diseases and major conditions, with an interpretation of real-world settings that predict disease and intercept pathology precisely, ideally preventing disease (Healthcare Information and Management Systems Society, 2019; GOV.UK, 2021b; GOV.UK, 2023b). PHM architects’ BM data training and analytic assurance, which promise to enrich each HPO lifecycle (Oddy et al., 2024; Afonina et al., 2023). Table 1 orchestrates the PHM mission through national initiatives while scaling regional capacity and adopting analytics capable of deploying truth for each citizen’s HPO pathological point of need throughout a lifetime (Institute of Biomedical Sciences, 2023).

**Table 1:** Population health management, national, regional, and citizen aims.

Population health management	National	For each citizen, improve healthcare delivery through enhanced data interoperability and innovation for scientific research integration.
	Regional	Integrated Care Boards (ICBs) expedite national *experts’* aims for local communities and citizens, specific to their ontology point of need.
	Citizen	Ensure everyone receives personalized care from their unique health profiles, leading to good health, well-being, and welfare.
Ontology risk stratification	National	Risk stratification optimizes healthcare plans and resources by focusing on prevention and early intervention for high-risk groups.
	Regional	Integrated Care Informatics Officers liaise on quality data, compute, and capacity to maximize data input for *expert* risk-stratification outputs.
	Citizen	Receipt lifetime predictors and appropriate care intercepts to prevent disease onset and target therapy to maintain a higher quality of life.
Ontology pathology segmentation	National	Tailor healthcare strategies to different ontology conditions as segments, improving effective and efficient healthcare services.
	Regional	Integrated Care Teams prioritize imminent risk in pathology segments to expedite *expert* intercepts, in the service of mental/physical wellbeing.
	Citizen	Benefit from individualized healthcare that foresees and affirms condition segments with target treatments for health and care management

### 2.4 Roadmap for public health and patient safety

Figure 1 shows the roadmap aligns analytics lifecycles with the national PHM mission to develop technology, achieving HIMSS maturity through data training and AI assurance, all stewarded by HEMSS. This approach ensures public and workforce alignment, promoting public health and patient safety in neighborhood HPO trials through procuring, integrating, monitoring, and evaluating AI tools in real-world settings, bridging healthcare gaps as scientific data themes and AI technology advance the PHM mission (Khan et al., 2024; Chan et al., 2024).

The DSIT, Genomic Medical Service, and Life Science sectors develop digital services to prioritize agreed-upon HPO projects that federate public health and patient safety learning over the next decade (Department of Science, Innovation and Technology, 2023; Department of Health and Social Care, 2022; GOV.UK and Industrial Strategy, 2025; Department of Health and Social Care, 2025). Technical and drug-funded workstreams are supported by capital funding that builds capacity through ICB, as fit-for-the-future funding equips society with PHM mission initiatives (GOV.UK, 2023c; England, 2025b; Labour, 2023). Population health management track inequalities stemming from the interplay between genomics and environmental factors, thereby personalizing HPO and improving health (GOV.UK and Office of Health Improvement and Disparities, 2023).

Figure 1 sets a roadmap that develops scientific data themes while adopting technology in the 10-year plan and consults on milestones for AI to deploy BM (Department of Health and Social Care, 2025; GOV.UK, 2024d; Farrugia et al., 2024). Commitment to the PHM mission across five points reshapes the primary care vision with genome-based personalized health capabilities for HPO needs in future health plans (Putman et al., 2023; Chu and Chung, 2024). As such, major projects that process capacity for PHM capabilities ensure real-world data enables responsible owners to develop predictors and adopt intercepts that translate public HPO health and patient BM safely (GOV.UK, 2024b; GOV.UK, 2023d).

## 3 Assess stakeholder engagement: evaluate the variables for data training

Figure 1 illustrates the stakeholder engagement in addressing variables in data training for neighborhood trials, with a focus on scientific themes, data pre-processing, labeling, and balancing, while mitigating bias. Real-world settings evaluate genomics, images, and social factor data, building on newborn screens for our adult future health by assessing features in lifecycle information (NHS England, 2024; Our Future Health, 2021). Our digital twin data are assessed in the following Sections: Section 3.1, “Primary Care for HPO Neighborhood Trials;” Section 3.2, “Data Alliances and Scientific Themes;” Section 3.3, “Data Structure and Cleaning in an Open-Source Ecosystem;” and Section 3.4, “Data Balance and Bias in Real-World Instances.”

### 3.1 Primary care for HPO neighborhood trials

Primary care services risk patient safety and public health due to increased workloads and poor informatics resources and processing, necessitating ecosystem-based scientific data themes to inform HPO stratifications (England, 2024). The benefits of technologies in these networks are identified in practice and population health variables and features for training data on disease segmentation and target therapy (Abdelaziz et al., 2024; NHS Wales, n.d.). Primary and social care services, trained on scientific data themes, support leaders who serve as HPO gatekeepers by extending datasets to aid in effective health decisions (Greenhalgh et al., 2023). A guide on informatics structure, capacity, and capability is essential for processing high-quality data to generate population health intelligence for management (NHS England, 2018).

Table 2 stratifies genome and health factor variables as images to segment pathology, while our primary care realizes HPO neighborhood trials. Our NHS confederation assesses our real-world data for AI in HPO, which integrates care with quality informatics and expert training that bridges gaps in primary care, public health, and patient safety (NHS Confederation, 2022; Orlowski et al., 2021). Service providers of genes, health determinants, and images analyze better outcomes as scientific data themes model HPO (Khoury and Dotson, 2021; Gurcan et al., 2015; Salgado et al., 2020). Stakeholders engage in neighborhood trials utilizing agile methods for digital health records that incorporate social determinants of HPO major conditions through data-driven training to predict and intercept pathology (Caicedo et al., 2024; Plans-Beriso et al., 2024). The diversity of a PHM mission is highlighted through points 1–5.

1. Genome patient engagement spans academic testing, research centers, nations, and continents to personalize neighborhood trials (Kreeftenberg et al., 2024). WGS pre-eXams for clinicians provide high-quality, clean data to identify variations for targeted therapies, including rare diseases, major conditions, and oncology (single cell and germline) (NHS England, 2020). Pharmacogenomics pre-eXams determine drug and dose efficacy, reducing adverse reactions (Palmer, 2022).
2. Imaging partners and providers build cooperatives, including academic imaging programs, commercial services, diagnostic clinics, research centers, and hospital radiology departments (Rong and Liu, 2024; NPIC, 2024). Imaging algorithms visualize BM across large datasets, improving primary care modalities from MRI to tissue scans that facilitate early disease detection and operate *in situ* with point 1 (Rong and Liu, 2024; NPIC, 2024).
3. Health determinants encompass a range of social, economic, and environmental factors that impact people’s well-being, such as income and vulnerability. Analyzing these factors enables comparisons between different areas (Public Health England (PHE), 2023). A statistical presentation that provides intelligence on metrics like unemployment within an ecosystem helps improve population health and reduce health inequalities. This approach may align with the principles discussed in points 1 and 2 (Office, 2024).
4. Biobank partnerships are global and include commercial, disease-specific, hospital-based, and national biobanks for population HPO projects, using science-themed data sources in points 1–3 for future BM delivery (De Souza and Greenspan, 2025). Biobank cooperatives further provide genomic classification, such as epigenetics, which has uncovered root causes of physical and mental perturbation that impact HPO as a primary care (Biobanking.com, 2024).
5. Life Science WGS pre-eXams will shape international academic centers, biotechnology firms, clinical research, government research, pharmaceutical companies, and authorities (Genomics England, 2024a). Life Science eXams utilize pre-processing and data annotation for personalized biotherapy, from gene editing to oligonucleotide interventions, which have the potential to cure diseases with target BM (Uddin et al., 2020; Egli and Manoharan, 2023).

**Table 2:** Assessment of genome or health factor variables to segment and image disease.

Genomic variables stratification	Human phenotype ontology segmentation by image	Health factor variables stratification
Rare diseases	Omics biological models	Social and economic
Physical conditions	Physiology, e.g., ECG	Health services quality
Mental health conditions	Tissue and blood images	Behavioral
Oncology	MRI and CT scans	Psychological
Drug optimization	Psychology/behavioral	Choice of GP
Nutrient optimization	Geospatial analysis	Personal lifestyle choices

### 3.2 Data alliances and scientific themes

Alliances’ scope encompasses data science themes, including nucleotides, images, and social factors, to stratify HPO risk in pathology segments. This approach aims to predict mental and physiological conditions, thereby personalizing the choice of intercepts for well-being (Ngiam and Lin, 2023; Panayides et al., 2020; Frank et al., 2020). The PHM mission is an analytic mindset for scientific themes, which aligns with HPO knowledge hubs and target therapy as public health screens value evidence-based care (NHSE, 2023b; Pirmohamed, 2023). Innovation expands from the UK Health Data Research Alliance, amalgamating healthcare and research organizations for the best ethical practices to accelerate BM for well-being improvements (UK Health Data Research Alliance, 2024). Global Genomic Health Alliances create policies for accessible platforms with standards and tools for data science community stakeholders, promoting responsible research sharing (Global Alliance for Genomics and Health, 2021).

Table 3 shows that the real-world benefits are unparalleled and equitable in a society that standardizes informatics in a national arrangement for PHM, with each person’s HPO profile considered for value-based care. A PHM strategy adopts an analytic mission in HPO policy for primary care action in neighborhood trials with value in health through informed consent and cybersecurity since data is the DNA of modern life (UK Parliament, 2024; GOV.UK, 2024a). Scientific genomes train digital twins for BM pre-eXams to predict health, while eXams align automatically for precise care (Katsoulakis et al., 2024). The HEMSS multidisciplinary teams develop agile methods and adopt other data science themes to sustain HPO primary care (Ma et al., 2024). Table 4 outlines the key concepts of science themes and AI technology, highlighting HPO and ML developments that align with the PHM mission for truth at the points of need through public inclusiveness with stakeholder engagement (Putman et al., 2023; Institute of Biomedical Sciences, 2023).

**Table 3:** Benefits from data alliances, scientific themes, and hpo primary care policy.

Benefits of international standard formats for ontology in a phm ecosystem		
PHM interoperability*	Data harmonization	Semantic clarity
Data lifecycle management	Education and training	Cultural sensitivity
Scalability	Research and development*	HPO operational efficiency*
Quality of care*	Regulatory compliance	Risk management*
Environmental health	Chronic disease management*	Emergency responses
Cost reduction in data use	Resource allocation	Citizen engagement*
Global collaboration*	Knowledge discovery*	Data security
Clinical support decisions *	Transparency and sustainability	Health equality
Public health surveillance *	Personalized care*	Artificial generative intelligence
Disease prevention *	Predictive analytics*	Precision analytics*

Genomics impact * in the PHM of HPO through BM.

**Table 4:** Scientific themes that feature HPO in machine learning developments.

Feature HPO	Machine learning developments
Family medical history ^G^	Cooperation with stakeholders to focus communities on ML
Treatment response indicators ^G^	Uphold ethics that favor citizen choice and privacy on PHM.
Predictive symptom clusters ^GS^	Choose algorithms for the nature or nurture of citizen ontology.
Health risk scores ^GS^	Training on population associations for parity
Chronic condition indicators ^GS^	Optimization in tuning hyperparameters for predictive power
Stress level indicators ^GS^	Validate & test diverse populace data for robust PHM ML.
Dietary habits ^GS^	Interpretability for practitioners and users with explainability
Social determinants ^S^	Integrate support decisions and public health neighborhoods.
Physical environment ^S^	Continuously monitor and maintain HPO safety evaluations.

G, Genomic science theme; S, Social or environmental science themes.

### 3.3 Data structure and cleaning in an open-source ecosystem

QA in data accuracy is a prerequisite for determining the informatics structure type for cleaning and benefits from a standard approach to open-source data processing, which labels and readies BM/HPO training.

#### 3.3.1 Data structure and storage

The PHM mission requires robust ecosystem interoperability and data storage while addressing the silo challenges with high data volumes, velocity, veracity, and variety (Ranchal et al., 2020). Genomics, imaging, and social determinant data are unstructured, semi-structured, and structured in standard BM/HPO applications for pre-processing (Salve et al., 2024; Praveen et al., 2017). Structured data is well-organized text that makes it easier to search biobanks or national databases and is typically stored in relational databases (Praveen et al., 2017). Semi-structured data falls between types and includes text documents, videos, and spreadsheets; it is stored in SQL or NoSQL databases, depending on the use case, with options such as BigQuery, Azure Cosmos DB, and DynamoDB (Praveen et al., 2017; Dallalba et al., 2022). Unstructured data, which lacks a defined format and schema, includes raw genome sequences and images and is often stored in NoSQL databases and biological infrastructures for HPO projects (Praveen et al., 2017; Dallalba et al., 2022). Life data drives efficient processing from genomic semi-structured types, as WGS bioinformatics pre-eXams are predictor infrastructures (Bagheri et al., 2019).

#### 3.3.2 Data cleaning

Employing rigorous data cleaning methods enhances the quality of informatics, improving the reliability and validity of research outcomes in the five-point PHM mission:

1. Genomics: Techniques align reference genomes and variant calling while cleaning data and addressing sequencing errors, missing values, and duplicate entries, “resulting in increasingly accurate interpretations” (Brlek et al., 2024).
2. Imaging: Image processing techniques and deep learning algorithms pre-process image informatics, including noise reduction, normalization, and artifact removal (Birkfellner, 2024).
3. Social Factors: Data imputation methods and normalization techniques are applied to clean social factors data, addressing missing values, inconsistencies, and biases in these challenging datasets (Balarezo et al., 2023).
4. Biobanks: Biobank data are cleaned by validating sample identifiers, correcting metadata errors, and integrating data from multiple sources to ensure consistency and accuracy (Shekhovtsov and Eder, 2022).
5. Life Sciences: Clinical trial data and laboratory results are cleaned using statistical methods and machine learning algorithms to remove outliers, handle missing data, and standardize measurements (Pilowsky et al., 2025; Ahmmed et al., 2023).

#### 3.3.3 Open-source processing as an ecosystem

**Figure 2:**
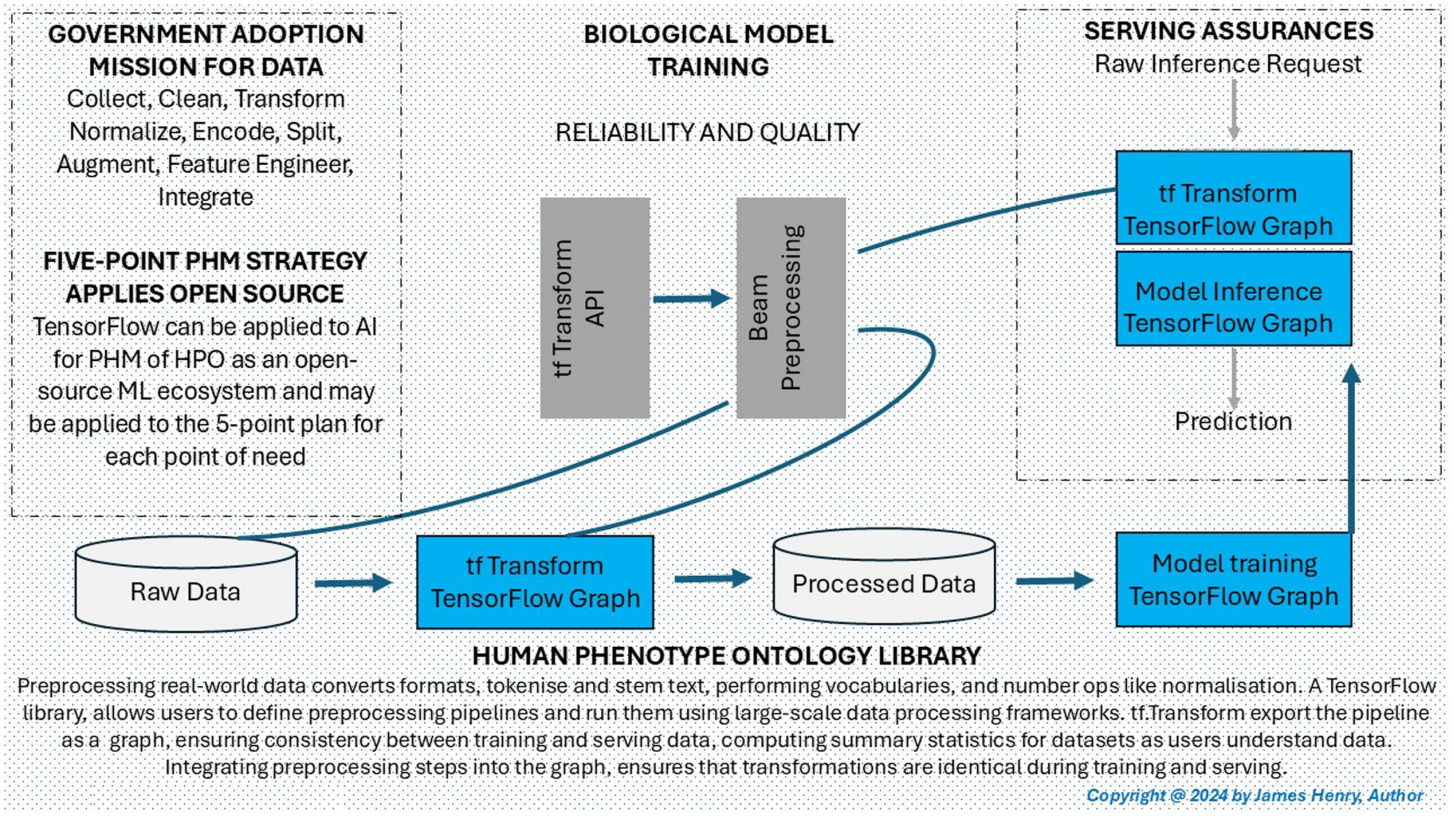
Population health an open-source ecosystem.

Figure 2 depicts an open-source ecosystem offering AI data pre-processing and an efficient pipeline. TensorFlow (TF) and PyTorch provide tools and libraries to train and deploy ML with DL, making them suitable for complex BM involving genomics, images, and health factor analysis for HPO (Python, 2024). Google TF is widely used on HPO projects due to its scalability within a genomic pre-eXam ecosystem, and it supports GPU/TPUs for high-performance computing to train on large datasets (Taylor-Weiner et al., 2019). PyTorch is more user-friendly and easier to implement, making it some researchers’ preferred choice for HPO projects with a dynamic computation graph. This flexibility in debugging and adaptability in processing and reinforcement learning tasks (Cursa, 2024) further supports its appeal. TF and PyTorch are potent tools within a PHM ecosystem for HPO applications across digital health records and medical imaging, with TF2.0 and the MONAI builds providing advanced capabilities (Bansal, 2021; Cardoso et al., 2022).

### 3.4 Data balance and bias in real-world instances

HPO balancing and EHR bias can affect the reliability of predictors and intercepts in real-world settings, so datasets used to train must accurately represent individual citizens and the population of interest (Ban Al-Sahab et al., 2023). BM bias is multi-sourced, encompassing data collection, labeling, and ML choice, with inaccurate personalization negatively impacting safety. In contrast, fairness identifies and mitigates bias by monitoring analytic solutions throughout the lifecycle (Mehrabi et al., 2025).

#### 3.4.1 Data balancing

In a PHM mission, data pre-processing normalizes and standardizes data for ML compatibility. This involves scaling numerical values, encoding categorical variables, and negating variations that impact performance: it transforms HPO into a standard format built on gene expressions in our environment (Colantuoni et al., 2002). Accurate labeling and resolving ambiguity for training BM annotate points for ML context, as patterns make accurate predictions (Fredriksson et al., 2020). WGS pre-eXam interpretation annotates label marker traits, while BM segment disease for eXam intercepts through pattern recognition (Bagger et al., 2024).

Data are split for PHM performance, with parameters trained to minimize errors and improve accuracy as the training set trains the HPO and a validation set tunes hyperparameters, whilst the test assesses the BM’s ability to generalize on the new data in a data balance flux of genomic scaling (Antonakoudis et al., 2020). It is imperative to evaluate dataset balance using appropriate metrics employed to provide the quantitative evidence for bias mitigation with fairness in model performance in before and after comparisons for reliable predictors and intercepts in real-world settings (Pagano et al., 2022).

#### 3.4.2 Bias mitigation in real-world settings

Table 5 structures bias types, their impact on PHM with HPO mitigation strategies, statistical metrics, and empirical results. Table 5 spans data, training, and user interface bias. It opens on an informative balance with demographic parity, equalized odds, and disparate impact ratio methods in mitigation techniques to significantly improve fairness in AI-driven comparative analysis for the PHM mission as a model (Shah and Sureja, 2024; Vidyadhar Sribala et al., 2024; Birdi et al., 2024; Schwartz et al., 2022). The table summarizes implicit or explicit notions of the emergent and social bias, which require PHM vigilance and governance, if not government oversight, in a competitive market where public health and patient safety classifications address a standard of fairness (Schwartz et al., 2022; National Institute of Health, 2024; NHS, 2024).

**Table 5:** Fair PHM of HPO Covers impact and mitigation with metrics and empirical results.

Bias types	Population health management bias impact and statistical metrics	Human phenotype ontology mitigating bias and empirical results
Measurement bias	Overestimation/underestimation skews outcomes by compromising accuracy	Rigorous validation and recalibration with statistical adjustments
	Demographic parity	Skewness is reduced by recalibrating for improved diagnosis with dataset accuracy.
Omitted variable	Spurious correlations or masked causation, misdirected interpretations	Ensure inclusion of relevant variables and capture delicate interactions
	Equalized odds	Enhance correlation accuracy by including relevant variables in the prediction.
Aggregation bias	Masks underlying variation and patterns via subgroups with inaccuracy	Balance the granularity and clarity to reflect diversity in aggregation insights
	Disparate impact ratio	Increased granularity balance subgroup data for improved predictive PHM
Sample bias	Introduces inaccuracy in population representation affecting HPO models	Use proper randomization techniques for data collection and use subsets
	Statistical parity	Improved population representation with proper epidemiology randomization
Algorithmic bias	Unfair decisions by the algorithm, even with unbiased training data	Utilize fairness-aware algorithms and incorporate constraints in training
	Fairness-aware algorithms	Increase algorithm fairness and reduce bias in the intercept chosen
Interaction bias	AI unintentionally learns and reinforces biases from user interactions.	Regularly monitor user feedback and adjust the model to mitigate bias.
	User feedback analytics	Reduce learned biases, monitor feedback to improve AI customer service
Population bias	AI prioritizes one group with inaccurate predictions for underrepresented groups.	Ensure diverse and representative training data to reflect the population.
	Population stability index	Improved group representation in diverse training data enhances PHM
Emergent bias	AI learns new biases during operation, leading to unfair decisions	Continuous monitoring of AI updates, detects, and mitigates emergent biases
	Bias detection algorithms	Emergent biases to fairer decisions use CCM to reduce biases autonomously
Social bias	Cultural attitudes and prejudices, leading to biased predictions	Address attitudes in data collection and promote inclusive practices
	Cultural bias metrics	Reduced prejudiced predictions address cultural attitudes through inclusiveness

## 4 Develop PHM architecture and adopt assured personal classifiers

The development of PHM architecture and the adoption of assured personal classifiers are essential for advancing public health. As depicted in Figures 1, 2, this work involves a digital twin of scientific data to deliver excellence in primary care (Katsoulakis et al., 2024) and an open-source PHM mission (Python, 2024; Taylor-Weiner et al., 2019; Cursa, 2024; Bansal, 2021; Cardoso et al., 2022). Initiatives support these efforts in the drive for digital identities, which provide a secure, ethical framework for giving individuals control and trust over their health data (GOV.UK, 2023e). This section outlines the private, secure, and ethical actions required to develop PHM architecture for WGS pre-eXam predictors and eXam target intercepts. Sections include 4.1 “AIDRS-AISI Authority Plans AI Architecture,” 4.2 “Develop an Explainable Model Vision and Language Performance,” 4.3 “Adopts National Generative Classification for Predictors with Intercepts,” and 4.4 “Adopts Guardrails and Ethics for a PHM Mission.”

### 4.1 AIDRS-AISI authority plan AI architecture

The PHM strategy for HPO builds a unified infrastructure aligned with the UK–US Memorandum of Understanding on AI architecture (UK.GOV, 2024). The AIDRS-AISI authority evaluates AI models and monitors HPO learning within a PHM mission that spans Federated Data Platforms (The AI and Digital Regulations Service, n.d.; AI Safety Institute, 2024; NHSE, 2023a). Key partners include Google Genomics for data storage and analysis, DeepMind for BM applications like ontology images and predictors, OpenAI to drive predictive health and precise care with advanced model transformers, and new startups to harmonize technologies within the primary care ecosystem.

#### 4.1.1 A comparative analysis of AI architecture

In Tables 6, a PHM mission is a comprehensive analysis of AI architecture for national HPO primary care excellence in the 10-year plan (Department of Health and Social Care, 2025; Department of Science, Innovation and Technology, 2023). It must first consider the weaknesses before the strengths in a PHM architecture against criteria that expedite the HPO primary care mission, such as scalability, interpretability, bias mitigation, and training time, which impact use cases at the point of need (Putman et al., 2023; Institute of Biomedical Sciences, 2023). PHM adoption would first consider DL perceptron feedforward neural networks, in a BM foundation offering vision and language processing with backpropagation (Lefkowitz, 2019). While Recurrent Neural Networks (RNNs) are suitable for time-series analysis, they are generally inappropriate for PHM due to process delays (Schmidt, 2019). LSTMs, a variant of RNNs, can address long-term dependencies in complex temporal sequences, but they are not ideal for high-throughput HPO analysis without significant scientific *transformation* (Hochreiter and Schmidhuber, 1997).

**Table 6:** AI architecture: a comparative analysis with metrics.

Criteria	CNN	LLM	Gen AI	RL	ViT	Hybrid AI
Scalability	High	High	High	High	High	High
Interpretability	Moderate to High	Moderate to High	Moderate to High	Moderate	High	High
Mitigate bias	Fair	Good	Good	Good	Very Good	Very Good
Training time	Moderate	Moderate	Moderate	Long	Moderate	Moderate
Accuracy	85%	88%	90%	83%	92%	94%
Specificity	83%	86%	88%	81%	90%	92%
Sensitivity	82%	85%	87%	80%	89%	91%
F1	84%	86.5%	88.5%	81.5%	90.5%	93%
Use cases	Biological model genomics and images	Health factors language and text	Review or generate diverse data types	Monitoring required potential redundancy	Future HPO imaging tasks	Combined applications for HPO classifiers

Table 6 reviews the proficiency of AI, commencing with Convolutional Neural Networks (CNNs) in genomics, diagnostics, and medical image processing for BM practicality (Baris Kayalibay and Jensen, 2017). Large Language Models (LLMs) excel in understanding text, translation, and conversational AI capabilities across HPO (Wang et al., 2024). Transformers offer superior performance in vision and language-based BM tasks (Shin et al., 2022). With all the attention needed, HPO models on transformer mechanisms capture long-range dependencies, achieving state-of-the-art BM classifications (Shin et al., 2022; He et al., 2022). In omics, Gen AI (GAN) leverages multimodal datasets and advanced algorithms to generate innovative solutions in a PHM mission for BM (Goodfellow et al., 2014). Reinforcement learning enables models to optimize decision-making over time (IEEE Xplore, 1998). Hybrid AI models combine multiple architectures in an all-inclusive approach for PHM of HPO as standard predictor and intercept classifiers (Das et al., 2024).

#### 4.1.2 PHM trade-offs with AI models

PHM expert approaches increasingly incorporate HPO, where hybrid AI models, particularly Vision Transformers and LLMs, demonstrate exceptional capacity to manage dependencies via self-attention mechanisms (Touvron et al., 2021; Wang et al., 2024). As detailed in Table 6, hybrid architectures offer superior metrics for intercept efficiency, but they also require substantial computational resources and infrastructure. This trade-off between performance and operational burden must be carefully evaluated. By integrating neural networks with transformer-based designs, hybrid models address the multifaceted demands of PHM while accounting for cost (GOV.UK, 2023c; England, 2025b; Labour, 2023). Their utility in realizing the genomic predictive health pre-eXam and eXam interventive classifiers underscores their strategic value, with the ultimate trade-off being the automation of clinical support decisions that enhance patient safety through agentic AI (Henry, 2025b; Henry, 2025c; Henry, 2025a).

#### 4.1.3 Structural and continuous control monitoring

Structural Monitoring (SM) and Continuous Control Monitoring (CCM) help identify the reliability of AI architectures during HPO domain development, whilst PHM diversity engages multiple types of SM and CCM, as provided in Table 7. Meanwhile, engineers and agile groups are developing bespoke continuous and non-HPO standardized digital PHM practices (van Ede et al., 2023). Transformer-powered experiences simplify disease observability in root-cause BM analysis from genome to protein structure (Hassan et al., 2022; Jänes and Beltrao, 2024). Therefore, by revealing key BM insights and streamlining workflows, AI transforms complex data to stratify risk and segment pathology, enhancing HPO through system engineering initiatives that make patient safety the norm, albeit requiring modern SM and CCM developments (Carayon et al., 2020).

**Table 7:** Structural monitoring and continuous control monitoring.

Tool	Type	Description
Intersect surveys	SM	Real-time data collection and analysis to sustain preprocessing
Advanced survey instruments	SM	Predictive maintenance, anomaly detection, and LiDAR 3D monitoring
Life science phenotype ontology	SM	Dynamic Retrieval Augmented Generation of Ontologies and LLM4Life
Population health management	SM	AI-powered healthcare and GenAI for care plan orchestration
Genetic algorithms	SM/CCM	Optimize population solutions and iteratively improve for SM and CCM resilience
BioRep monitoring and control systems	CCM	Provides control of operating parameters in biobanks, ensuring the integrity of biological samples
Cloud LIMS Biobanking	CCM	Manages patient data for compliance with guidelines, maintaining data integrity and security
Governance, risk, and compliance solutions	CCM	Integrates solutions for real-time insights into control activities and support compliance or audit readiness

SM, Structural Monitoring; CMM, Continuous Control Monitoring.

### 4.2 Develop an explainable model vision and language performance

Figure 1 expands the U.K.’s strategic framework for global health innovation, using HPO as a foundation for explanation in conventional medicine. This approach aligns with a PHM model powered by HPO-AI as Explainable AI (XAI) techniques that reform BM for pre-symptomatic medicine (GOV.UK, 2023f). The U.K.’s science and technology strategy, led by the DSIT, emphasizes transparent and accountable AI decisions and regulation to ensure patient safety, data privacy, and public trust in PHM applications, particularly in predictive and intercept classifiers (Department for Science, Innovation and Technology, 2023). In the world context, the European Union’s AI Act outlines transparency obligations for ecosystem deployers (European Union, 2024), while the United States NIST develops properties for AI trust (Phillips et al., 2021). International explainable high-performing analytics begin with the WGS of germline and single-cell data, which form the basis for BM genomic predictive health pre-eXam with precise care eXams in the HPO transformation (Maqsood et al., 2024; Henry, 2025b).

#### 4.2.1 Visual transformers in oncology

Visual transformers (ViTs) enhance predictive vision and improve precision assessments, enhancing interpretability and efficiency. The importance of oncology ViTs stems from WGS germline and single-cell genomic GANs/CNNs with synthetic data that mimic informatics, seeking optimal predictors in deeper learning methodologies (Thakur et al., 2024; Latha et al., 2024). ViT enhances capabilities with self-attention layers, which capture complex relations with techniques like SPARSE attention to improve effectiveness in real-time applications (Chen et al., 2021). SPARSE attention involves selectively focusing on a smaller subset of relevant data points, reducing computational complexity and improving the model’s efficiency (Chen et al., 2021). CNN interpretability, and even more so ViTs, spans BM to HPO, exemplified in breast cancer, where the optimal metrics investigated digitally enhance classifications and exemplify the Pre-eXam and eXam concepts (Thakur et al., 2024; Latha et al., 2024). Visual transformers represent a significant advancement in AI to capture complex relationships in large datasets: self-attention enables them to focus on key elements of an image, making them more effective (Dosovitskiy et al., 2021).

#### 4.2.2 Transforming the language in ontology

NLP techniques have evolved from basic text processing and rule-based systems to advanced LLMs with the advent of agile DL methods like Word2Vec, which capture semantic meaning from large text corpora (Mikolov et al., 2013). Developing transformer architectures has advanced the field of ontology, enabling LLMs to understand complex data and generate human-like text (OpenAI, 2023). BM applications with multimodal data in a safe space combine text with images and other health-determinant data types for population health risk stratification and HPO segmentations to identify true predictors and intercepts, as demonstrated in a framework for biomedical applications (Wu et al., 2025). NLP has brought remarkable breakthroughs in LLM-driven success for U.K. Biobank data types, while genomic Pre-eXams support predispositions to mental and physical HPO in personalized plans, which benefit from policy (UK Biobank, 2024a; Henry, 2025c). LLMs are trained to map text descriptions of research, health records, symptoms, diagnoses, and social determinants, as defined in literature reviews, to provide digital twin support for accurate eXam intercepts in a global stewardship for reform with HEMSS (Dennstädt et al., 2024; Henry, 2025a).

By transforming the language in ontology, the author contrasts SHAP values that offer information about how each feature contributes to a model’s output with LIME, which focuses on approximating the model locally around a prediction to explain it (Shaikh et al., 2023). SHAP values offer consistency and accuracy in cooperative theory, making them more stable across different datasets and models (Shaikh et al., 2023). Nevertheless, LIME is highly flexible and can be applied to any model, providing local fidelity to specific predictions, but it might lack global consistency (Shaikh et al., 2023). SHAP is well-suited for understanding the importance of features across the entire dataset, while LIME explains individual predictions in a highly interpretable manner (Shaikh et al., 2023). Technologies like SHAP and LIME underpin unified approaches, assigning important values to data features for interpretability by identifying predictors or intercepts, thus improving HPO accuracy (Shaikh et al., 2023).

In the context of Table 6, SHAP and LIME can be effectively applied to CNN, LLM, Gen AI, ViT, and hybrid AI models to provide insights into scientific data feature contributions and local approximations of model predictions. LLMs train cross-reference data across primary care to identify socio-environmental factor impact on ill health (McCoy et al., 2024). The interpretability of LLM attention weights provides insight into which part of the model’s input text it focuses on and whether it is attention-seeking (Vashishth et al., 2019). While LLMs may interpret a high incidence of pathology with metrics, there remain conjectural findings that require a PHM ecosystem for HPO action as the primary care (Ho et al., 2024). An evaluation of LLM performance that focuses on metrics addresses challenges by ensuring reliable and accurate predictions in PHM applications with digital twins (Hu and Zhou, 2024; Katsoulakis et al., 2024).

### 4.3 National generative classification for predictors and intercepts

Following COVID, BM is entering a transformative decade, driven by national analytics of health trajectories that enable digital integration for generating predictor and intercept classifiers (Mauer et al., 2021). For example, the concept of self-attention, which segments cellular and molecular mechanisms and mitigates individual health risks using synthetic data for medicines, offers more than theoretical promise and provides actionable insights. This approach is exemplified by a study on fibrosis, which demonstrates how such a data-driven model can inform targeted medicine development through X-AI (Henderson et al., 2020).

Table 8 instance genomic health predictive Pre-eXam (P) and precision care eXam intercepts (I) as a unique classification for each citizen (X = Gen AI), with agentic AI autonomous linkage (Henry, 2025b; Henry, 2025c; Henry, 2025a) projected over the coming decade in the development of life sciences (Department of Health and Social Care, 2025; GOV.UK and Industrial Strategy, 2025). National Generative AI aligns the Five-Point PHM mission as an ecosystem with Sections 4.3.1 Interpretable BM and 4.3.2 Transparent HPO, which would prepare our society for 4.3.3 The Trustworthiness of PHM.

**Table 8:** Real-world setting for predictors and intercept classifications.

Predictor- intercept classification	Generative AI transparency in the real world with the human phenotype ontology, the primary care
Rare Diseases ^P^	Address ethical and legal data use for ontology use case ^P I^
Gene Therapies ^I^	Focus on the design of proactive management by the practitioner and citizen ^P I^
Teen Mental Health ^P^	Coordinate multidisciplinary roles from development to adoption ^P I^
Assess Mental Health ^I^	Semantic interoperability and traceability services for sector ^P I^
Adult Major Condition ^P^	Integration know-how of ecosystem data sources for HPO models ^P I^
Personalized Plans ^I^	Impact analysis of ^P^ in relation to ^I^ is assessed for population groups
Elderly Multimorbidity ^P^	Data-driven decisions from the PHM ecosystem to the HPO models ^P I^
Review Polypharmacy ^I^	Risk stratification ^P^ and segment disease groups ^I^ for effective PHM
Nutrient/Pharma-omics ^P^	Neighborhood clinics engage and educate the public on outcomes ^P I^
Lifelong targets ^I^	Feedback loops, reinforcement learning, and continuous improvement ^P I^

^P^, Predictors; ^I^, Intercepts.

#### 4.3.1 Interpretable biological modeling

Interpretable BM with autoencoders is achievable as Gen AI trains the predictors and intercepts, while the latent space reveals clusters of characteristics to subtype diseases and target therapy (Doncevic and Herrmann, 2023; Farhadyar et al., 2024). The quality of the synthetic data for foundational BM is assessed by comparing its statistical properties, such as the distribution of phenotypes and allele frequencies, with those of actual patient data, ensuring it is representative and reliable (Goshisht, 2024). Revolutionizing personalized well-being with Gen AI actioning our digital twin through the predictive Pre-eXam, which instantiates the U.K. Newborn Generation Study in a standard approach to targeting 200 rare disease eXams (Katsoulakis et al., 2024; Genomics England, 2024b). Gen AI models trained on synthetic data offer faster, accurate, tailored therapies, improving outcomes through personalized treatment simulations based on the WGS-BM, predicting likely responses to different therapies, and identifying personal intercepts that commence with bioinformatic pharmacogenomics (Center for Drug Evaluation and Research, 2019; Center, 2024). The Pre-eXams-eXams Agentic AI is an interpretation of BM truth for conventional medicine to expedite value-based care that becomes the norm in a simplistic major condition strategy (Henry, 2025b; Henry, 2025c; Henry, 2025a; GOV.UK, 2023b).

#### 4.3.2 Transparent human phenotype ontology

Accessing the phenotype Pre-eXam requires consent to open a transparent health journey, enabling Gen AI to provide timely, targeted, and practical classifications based on polygenic risk scores, pleiotropy, and socio-environmental factors that affect the probability of succumbing to a major condition (Loika et al., 2020). In the PHM mission, the ambiguous predictors and intercepts become clear, requiring proactive HPO designs and coordinating multidisciplinary roles that engage the public on outcomes in a new era of semantic interoperability with Gen AI (Midlej and Medeiros, 2017).

Gen AI eXams have ethical, legal, and social implications when based on predictions as a transparent primary care, which differentiates multifactorial diseases for sound interventions (Chapman, 2022). Gen AI empowers polygenic risk scores by analyzing vast amounts of genomic data to assess disease susceptibility, improving outcomes when early interventions reduce health burdens (Cognizant, 2024). Gen AI also integrates socio-environmental factors to better understand their impact on mental health conditions, with intercepts that span community well-being (Kambeitz and Meyer-Lindenberg, 2025).

Furthermore, greater transparency with Gen AI and public value is required from biopharma companies, which oversee the factors influencing biomarkers and treatment responses (Spencer et al., 2025). Rapid integration into public health infrastructure requires ethical guidance to manage data privacy and tech challenges for universal advancement in spatial risk prediction, health surveillance, disease forecasting, and diagnosis (Olawade et al., 2023). Gen AI enhances HPO efficiency and automates primary care tasks through rapid, transparent integrations in standard classifiers, facilitating clinical support decisions (Rahmanti et al., 2024).

#### 4.3.3 Trustworthiness of population health management

Trustworthiness in PHM is directly linked to the fairness in genomic health and explainability of AI, which are central to the U.K. national digital strategy (Department of Science, Innovation and Technology, 2023). Table 9 aligns PHM with the HIMSS maturity, developers, while HEMSS principals steward the adoption of classifiers to accelerate reform in a trusted ecosystem of fit life cycles in analytics. In another article, “Global Reform: PHM Higher Expert Medical Science Safety,” the author explains how HEMSS stewardship simplifies the development of classifications within a transformative ecosystem that improves evidence-based PHM and ultimately enhances our quality of life (Henry, 2025a). This trust in PHM is proposed by the author in a United Nations Scientific Policy Brief on Sustainable Development Goals (United Nations, 2025), as well as a WHO initiative for health and well-being in traditional medicine.

**Table 9:** AISIAIDRS Authority on HPO Primary Care with HIMSS and HEMSS Proposed.

HIMSS Voluntary: classifier development	HEMSS Authority: classifier adoption
Health informatics and management system society maturity levels 0–7	Higher: Hybrid Infrastructures Classifications
Infrastructure readiness and adoption model	Expert: agile group developments develop classifications
Adoption model for the application of AI in medical and radiological management	Medical: practitioner and biopharma stakeholder engagement—adopt classifiers
Electronic medical record adoption model	Science: multi-omics, images and health factor open data themes to classify
Continuity of care record adoption model	Safety: public health, patient safety and parity public inclusiveness—adopt classifications

### 4.4 Guardrails and ethics adoption in a national PHM program

The U.K. government is establishing a comprehensive and ethical framework to integrate data into the PHM mission for public healthcare, with clear guidance on standards (GOV.UK, 2025). This initiative would uphold an individual’s right to choose a WGS pre-eXam and consent to access BM for accurate disease predictors, with permission to receive a precise biopharma eXam, wherein AI ethical standards and guardrails are in place (Halamka, 2022). The disruptive nature of AI technology presents an opportunity to create a standard ecosystem for BM, which impacts health and supports clinical decisions, whilst experts align a moral approach to PHM with legality in HPO perspectives (Corfmat et al., 2025). The HRA and NIHR roles ensure that the PHM program and HPO projects adhere to ethical approvals and governance, necessitating streamlined regulation to address emerging AI challenges (National Institute of Health and Research, 2024). National aims include AISI evaluations and AIDRS authority with the guardrails and ethics depicted in Table 10 to showcase the national PHM of HPO predictors and intercepts.

**Table 10:** National guardrails and ethics justify a PHM Mission.

National population health management mission ethics and guardrails	
Robust governance is a priority to oversee ^GSPI^ data privacy	Clear governance to oversee Artificial General Intelligence
Privacy design is advocated by the Department of Innovation, Science and Technology	Adoptive Trusted Research Environments provide a safe ^PI^ space via verified researchers
Adopt Trusted Research Environments as secure access to ^GS^ with guardrails	Modernize software infrastructure for safe ^PI^ through Artificial General Intelligence
Assure ^GS^ data minimization, as per the UK Data Use and Access Bill	Improve interoperability ^GSPI^ for the seamless PHM of HPO with BM
Action Semantic interoperability ^GSPI^ data and ethical use	Engage communities on ^GS^ with societal evidence-based value on ^PI^ trust
Engage communities on ^GS^ for trust in ^PI^ at each point of need with a classified ecosystem	Enhance ^GS^ curation on accurate ^PI^ management, which is explainable and interpretable
Enhance ^GSPI^ security from unauthorized use with national cybersecurity strategies	Enhance ^GS^ transparency for safe ^PI^ research, which is validated as a primary care
Advanced consent for ^GSPI^ shares per the UK Data Access and Use Bill	Promote collaboration and method shares across regions and nations
Regular audits for privacy and ^GSPI^ data protection	Continuous learning for Populace Health ^PI^ points of need with Gen AI

G, Genomic science theme, ^S^, Social or environmental science themes, ^P^, Predictors and ^I^, Intercepts.

#### 4.4.1 Human phenotype ontology guardrails

HPO guardrails individualize choice in the ethical use of real-world data in a PHM mission, which actions informatics: provenance, purpose, protection, privacy, and preparation (Segalla and Rouziès, 2023). The NHS Resolution on fairness and parity aims to develop a PHM ecosystem that manages performance in conjunction with workforce welfare and support, while also understanding the practitioner (NHS Resolution, 2025). Table 5 is revisited to establish guardrails that mitigate bias in data, algorithms, and user interfaces, ensuring national responsiveness and fairness in responsible AI ownership. Robust strategies for quality data, assured AI, and governance are essential for a lifecycle that develops disorder truth in Pre-eXam points of need (Ghalavand et al., 2025; Institute of Biomedical Sciences, 2023; Henry, 2025b).

Responsible UK genomic AI features dimensions for life science eXam developers and target therapy adopters, as the NICE-AIDRS commission HPO classifiers as the primary care (Bernasconi, 2021; Henry, 2025b; Putman et al., 2023; GOV.UK and Industrial Strategy, 2025). Global regulatory frameworks for the PHM mission are vital for ethical AI in real-world settings with EU guardrails for data protection and privacy (European Union, 2016). The U.S. Health Insurance Portability and Accountability Act also sets the standard for sensitive patient data protection (HIPAA Journal, 2019). Moreover, ISO 27001 provides a framework for managing information security risks in any organization (ISO, 2022). Compliance with GDPR, HIPAA, and ISO 27001 across society protects citizen GSPI data through a multifaceted approach, as detailed in Table 10, which provides guardrails within a Pre-eXam and eXam framework and potential for international policy and guidance (Henry, 2025c; Henry, 2025a).

#### 4.4.2 Ethics on populace genomics

The U.K. initiated a Genomics Ethics network to study the ethical and social issues of integrating genomics into routine clinical care (Gaille and Horn, 2021). An individual’s privacy should be respected when their genomic information is used for research and clinical applications, balancing progress with privacy (National Human Genome Research Institute, 2013). However, the four main ethical principles of beneficence, non-maleficence, autonomy, and justice do not fully resolve a person’s right to the best digital healthcare (Varkey, 2020). Governing AI in public health requires an HPO policy to ensure ethical public health and patient safety and to advocate for a call to action (Henry, 2025c).

An HPO transformation authorizes BM evidence and considers diversity, public choice in genome use, and bias negation in a Pre-eXam ecosystem (Henry, 2025b). Integrating genomics shifts the one-size-fits-all approach to negate diversity and target effective therapies, informed by policy for transformation to HPO eXams, which thereby even out parity (Henry, 2025c; Henry, 2025b; Bentley et al., 2017). However, transparency in how genomic data is used, who has access, and how decisions are made builds public trust when personalized wishes are respected (Szego et al., 2013). Structuring reform from pangenomes over a decade provides value-based care in an ethical era of BM (Taylor et al., 2024). Public inclusiveness balances the ethics of individual choice to access HPO as their primary care while respecting others’ privacy rights not to engage (Andrade, 2024).

## 5 Fit lifecycles in analytics adopt the PHM mission

**Figure 3:**
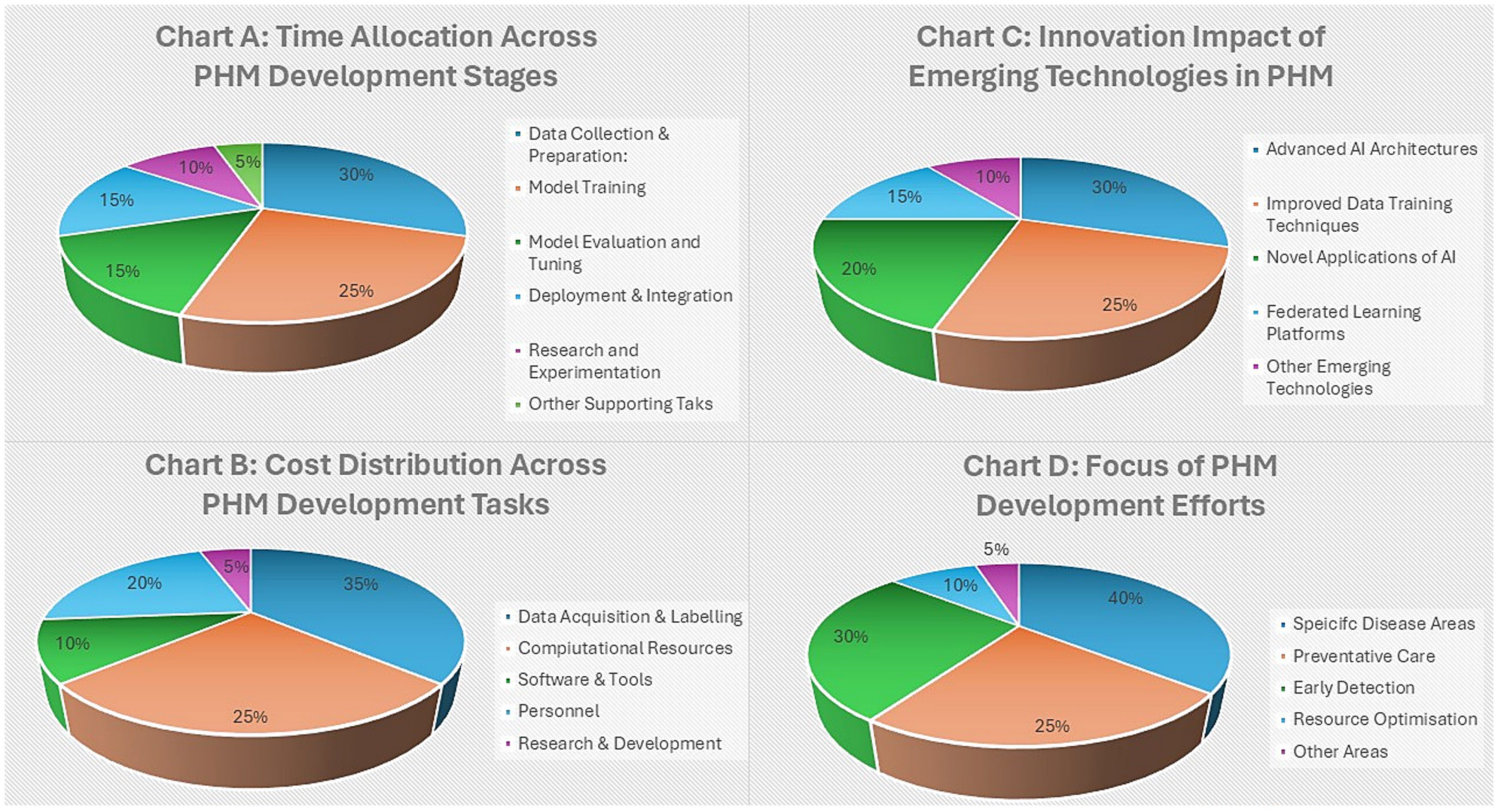
Population health management, the developmental dependencies.

The Science and Technology manuscript discussion on fit lifecycles in analytics draws inspiration from Life Science Healthcare Goals and the National AI Strategy, aiming to enhance productivity and service delivery in public health, patient safety, and parity (GOV.UK and Science and Innovation, 2024; UK Government, 2021; GOV.UK and Industrial Strategy, 2025; Department of Health and Social Care, 2025). The PHM mission is realized in an independent AI action plan with a series of adoption missions that expand the ecosystem through HEMSS’s ability to scan and scale for pilot classifiers (UK Government, 2025; Henry, 2025a). The Government DSIT plan for AI sets 10 principles for excellence in infrastructure development, which the author aligns with the HPO-BM assessment as the primary care (Government Digital Service and Department for Science, Innovation and Technology, 2025; Department of Science, Innovation and Technology, 2023; Henry, 2025b). The PHM mission is illustrated:

- Figure 1 provides a PHM roadmap of HPO and BM to steward data training and explain AI.
- Figure 2 provides an open-source genomics ecosystem for robust HPO pre-processing.
- Figure 3 provides PHM dimensions for deployment across primary care network integration.

Government guidance and an independent report continue to excel in QA recommendations from Section 1.1 point 6 as the author adopts robust technology to engineer PHM reform in a government proposal (UK Government, 2025; Government Digital Service and Department for Science, Innovation and Technology, 2025). A significant limitation is that while authorities address capacity, they fail to idealize the PHM mission with standard HPO capabilities or to inform on a BM norm in transformation. To resolve this, HEMSS stewards the HIMSS for pre-eXam genome health predictors and eXam digital twin therapies (Henry, 2025a; Katsoulakis et al., 2024). The manuscript debates in Sections 4.1–4.4 build on an AI engineering mindset for stakeholder engagement while prioritizing public trust in secure and safe futures with untold benefits to predict and diagnose early and provide accurate life intercepts (UK Parliament, 2024; GOV.UK, 2024a).

### 5.1 A national plan for fit lifecycles in analytics

The adoption of the PHM mission is a 10-year plan primarily focused on the genome and socio-environmental data, which we envision will be achieved through Hybrid-Gen AI agile methodologies for public health and patient safety (Department of Health and Social Care, 2025; Department of Health and Social Care, 2022; Public Health England, 2017). Table 11 presents the development of PHM and HPO adoption, which classifies scientific data themes and AI technologies for WGS predictive health Pre-eXam and eXam target therapy or lifestyle intercepts in the transformation of BM as our primary care (Henry, 2025b).

**Table 11:** Fit lifecycles with genomic health pre-eXam and precise care eXam intercepts.

Develop a future of PHM: science and technology advancement	Adopt HPO: skill sets required
Early intervention and prevention efforts in PHM result in better health outcomes as AI aligns with pre-eXam/eXam personalized healthcare principle 2	*Population Health Initiatives*: HPO Policy Writer, Bioinformaticians, Healthcare Data Analysts, Public Health Scientists
Tech suppliers provide digitally enabled solutions, improving patient engagement and healthcare delivery through classification	Digital health solutions: Software Engineers, Data Analysts, UX/UI Designers, Cybersecurity Specialists
Quantum computing processes complex health data, reforming diagnostics and treatment plans to scale predictors and intercepts	Quantum intelligence: Quantum Computing Researchers, Data Scientists, Bioinformaticians, Mathematicians
Cooperative ML across multiple institutions shares data and enhances data privacy/security in Pre-eXams/eXams	*Federated learning*: Machine Learning Engineers, Cybersecurity Specialists, Data Engineers, Privacy Experts
Engaging to accelerate the production and availability of advanced therapies relies on pre-eXam for the bio eXam	*Cell and gene therapy development*: Biomedical Engineers, Cell Biologists, Clinical Researchers, Regulatory Affairs Specialists
Cooperative advancements in treatments and therapies benefit both patients and healthcare providers with genomics insights	*Public/private sector initiatives*: Genomics Researchers, Healthcare Policy Advisors, Collaboration Coordinators, Data Analysts
Using third parties to enhance real-world evidence, leading to more accurate and effective treatment plans with a baseline of our DNA	*Real-world evidence and enhancement*: Data Scientists, Clinical Researchers, Bioinformaticians, Biostatisticians
Advanced Gen AI improves patient communication and data analysis to personalize each citizen’s healthcare in pre-eXams/eXams	*GPT-5 and Gemini 2.0*: AI Researchers, Data Scientists, Software Engineers, Healthcare Communication Specialists

Figure 3 Chart A, provides insights into PHM time allocation to develop the 10-year plan, which includes the investment required for a robust infrastructure in Table 11. Understanding where time is spent in PHM will result in better resource allocation and ensure that the mission is achieved efficiently through the principles of the Technical Guidelines (Government Digital Service and Department for Science, Innovation and Technology, 2025; Department of Science, Innovation and Technology, 2023). For instance, if data collection or preprocessing consumes much time, streamlined strategies are prioritized, as shown in Figure 2, with a commitment to a singular open-source ecosystem (Python, 2024; Taylor-Weiner et al., 2019; Cursa, 2024; Bansal, 2021; Cardoso et al., 2022).

The AI PHM opportunities represent an adoption mission aimed at building sufficient, secure, and sustainable infrastructure, with further progress sustained through investments in computing power and the development of primary care well-being (UK Government, 2025). Unlocking data scientific themes as assets in the public and private sectors is the PHM mission, requiring strategic planning and public trust in BM classifications to align HPO adoption with a joint science and technology mission for public well-being through policy (UK Government, 2025; Henry, 2025b; Henry, 2025c).

#### 5.1.1 Integrated care systems and ICB chief informatics officers

Government Principle 7 emphasizes openness and collaboration (Government Digital Service and Department for Science, Innovation and Technology, 2025). The PHM mission aims to support open science themes in robust technical infrastructure by integrating HPO policy for truth at the point of need (Python, 2024; Taylor-Weiner et al., 2019; Cursa, 2024; Bansal, 2021; Cardoso et al., 2022; Institute of Biomedical Sciences, 2023). In Tables 1, 4 ICBs coordinate capacity for the PHM of scientific data with healthcare AI services to ensure comprehensive and continuous care. CIOs oversee advanced health informatics solutions, enabling seamless data flow and real-time decision-making to personalize HPO benefits in Tables 2, 3.

Government Principle 6 states, “Use the right tool for the job,” with the importance of selecting appropriate technologies for effective HPO transformation as our primary care (Government Digital Service and Department for Science, Innovation and Technology, 2025; Henry, 2025b). Challenges with tools include high costs associated with AI infrastructure, computational power to process large volumes of data, and the constraints imposed by existing healthcare policies and regulations (UK Government, 2025). The implementation of HPO vocabulary is necessary for a PHM mission with real-world WGS predictors and digital twin intercepts adopted in national classifiers (Putman et al., 2023; Department of Health and Social Care, 2022; Katsoulakis et al., 2024) as shown in Table 8. National AI comparison and monitoring are provided in Tables 6, 7.

#### 5.1.2 Overcoming public concerns in PHM

Government Principle 2, emphasizing the lawful, ethical, and responsible use of AI, means responsible owners should prioritize guardrails and ethics for HPO primary care, ensuring data protection, fairness, and transparency. Nevertheless, nations differ in their approaches to AI and what we mean by global health explainability (Department for Science, Innovation and Technology, 2023; European Union, 2024; Phillips et al., 2021). This requires international HEMSS stewardship for global reform (Henry, 2025a). Public concerns are alleviated through justified data governance, privacy design, and advanced consent initiatives detailed in Table 10. Fit citizen lifecycles are developed through early intervention, federated learning, and advanced AI solutions (Deshprabhu, 2023; Ur Rasool et al., 2023; Rabbani et al., 2025; Groza et al., 2024; Dhade and Shirke, 2024; Stamatis et al., 2024), as outlined in Table 11.

Government Principle 3 emphasizes the importance of robust security, data privacy, and bias mitigation, in which a secure and resilient PHM mission necessitates transparent governance, privacy design, data minimization, and cybersecurity strategies to protect Genomics and Social factor data (GS) for Predictors and Intercepts (PI). As detailed in Tables 2, 3, 5, 10, these measures ensure that genomic and health factor variables are accurately assessed and community trust is built through ethical and transparent practices, safeguarding sensitive information against cyber threats and unauthorized access (UK Parliament, 2024; GOV.UK, 2024a).

Other challenges in a PHM mission include the use of public funds and financial investments for efficient resource use and sustainability, which involves exploring innovative partnership financing (GOV.UK, 2023c; England, 2025b; Labour, 2023; Kreeftenberg et al., 2024), which prioritizes the dimensions detailed in Figure 3. Advancements in cloud/edge computing mitigate power constraints by distributing the data processing load across nodes, reducing reliance on a centralized infrastructure, with a common approach to a national open-source ecosystem (Python, 2024; Taylor-Weiner et al., 2019; Cursa, 2024; Bansal, 2021; Cardoso et al., 2022) depicted in Figure 2 for genomics and images.

### 5.2 AIDRS stewardship and AISI evaluation of population health management

AIDRS/AISI governance and evaluation are expected to oversee the PHM mission on federated data platforms (The AI and Digital Regulations Service, n.d.; AI Safety Institute, 2024; NHSE, 2023a). In Section 2, the author assesses stakeholder engagement and data training to address real-world biases in HPO neighborhood trials, which underpin the AIDRS development for BM/HPO fairness (Pagano et al., 2022; Shah and Sureja, 2024; Vidyadhar Sribala et al., 2024; Birdi et al., 2024; Schwartz et al., 2022) in Table 5. In Section 3, the AIDRS/AISI authority could conduct a comparative analysis of AI architecture for HPO monitoring in the PHM mission proposed by the author (Henry, 2025b; Henry, 2025c; Henry, 2025a) towards ideal aims (NIST, 2024), as detailed in Tables 6, 7, which also monitor BM/HPO performance.

Figure 3, Chart D, illustrates AIDRS governance and AISI evaluation in the PHM development efforts, which address domains for successful HPO implementation and impact across different areas (The AI and Digital Regulations Service, n.d.; AI Safety Institute, 2024; NHSE, 2023a). Stakeholders, when consulted, identify gaps and ensure resources are stewarded effectively and efficiently for research and governance in evidence-based practice (Darzi, 2024; Khan et al., 2024; Chan et al., 2024). Meanwhile, there are many discussions, such as whether disease negation receives less focus than treatments. In that case, a need to reevaluate priorities underpins the genome health Pre-eXam, which aims to eradicate unnecessary pathology eXam dependencies globally (Henry, 2025b; Henry, 2025c; Henry, 2025a).

The PHM opportunities represent a science adoption mission, with technology supported by the private sector to integrate algorithms, thereby boosting productivity and innovation for BM realization with a radical thought that markets may resist standardization (UK Government, 2025). In a PHM mission, scaling AI adoption across the economy requires concentrating on HPO vocabulary for well-being in eradicating cumulative rare diseases and segmenting major disorder subtypes to target therapy (GOV.UK, 2021b; GOV.UK, 2023b). The diversity in medical terms requiring HPO annotation will contribute to socioeconomic success through the adoption of BM in primary care as a phenotypic norm (Putman et al., 2023).

#### 5.2.1 Governance through AISI/AIDRS authority

An AIDRS/AISI authority evaluates and governs safe and effective AI healthcare technologies using standards and guidelines to develop, deploy, and assess BM and patient impacts, which commence with Sonnet 3.5 ideals (NHS, 2024; NIST, 2024). However, as the PHM mission expands, the AISI/AIDRS evaluates data quality and integrity and reevaluates AI infrastructure, monitoring, and metrics with medical science academic experts in their HPO/BM using federated data (The AI and Digital Regulations Service, n.d.; AI Safety Institute, 2024; NHSE, 2023a). Indeed, for continuity and public health, the AIDRS authority must recommend HPO adoptions and justify X in the WGS Pre-eXam and eXam intercepts with Gen AI [X], which symbolizes validation and transformation through policy (NHS, 2024; Henry, 2025b; Henry, 2025c).

Government Principle 5 emphasizes the technical importance of managing the entire AI lifecycle through rigorous validation, continuous monitoring, and feedback mechanisms, thereby identifying and addressing delays in BM or negating HPO hallucinations (Government Digital Service and Department for Science, Innovation and Technology, 2025). The PHM mission integrates icteric fit lifecycles in analytics through AIDRS oversight of robust BM/HPO research (UK Government, 2025; NHS, 2024). AIDRS/AISI regulation, commission, and governance support secure and explainable decision-making (UK Government, 2025; The AI and Digital Regulations Service, n.d.; AI Safety Institute, 2024), while the author reminds society that it requires a global stewardship of medical science and technology for true reform (Henry, 2025a).

#### 5.2.2 Implementation of AI-driven PHM for HPO policy

Government Principle 8 recommends collaborating with commercial colleagues from the outset, given that AI-driven PHM necessitates investments in infrastructure, workforce training, and regulatory alignment, as outlined by the author in Section 4.1 (Government Digital Service and Department for Science, Innovation and Technology, 2025). Healthcare professionals are trained to use AI tools effectively, interpret BM insights, and make informed decisions for the AIDRS to authorize agile group developments (NHS, 2024). Primary care HPO requires hardware, including high-performance computing systems, secure data storage solutions, and specialized AI software in national evaluations (The AI and Digital Regulations Service, n.d.; AI Safety Institute, 2024).

Government Principle 10 emphasizes the importance of integrating principles into the organization’s policies and ensuring the correct assurance (Government Digital Service and Department for Science, Innovation and Technology, 2025). The author believes this manuscript has the potential to provide insight into AIDRS/AISI, Primary Care Network, and Public Health HPO policy with WGS Pre-eXam and eXam classifications (Henry, 2025c; Henry, 2025b). As detailed by the author in original works (Subsection 2.1, point 6), the goal is an ecosystem and to steward what is practical and efficient within the PHM five-point mission, which installs resistance to reform.

### 5.3 Future NHS HPO learning with new technologies

Government Principle 1 emphasizes knowing what AI is and its limitations (Government Digital Service and Department for Science, Innovation and Technology, 2025). At the same time, the author constantly reviews the future NHS initiatives in Table 11 and supports the PHM mission efficiency with scientific data themes pre-processed through Section 2 of the manuscript. HPO learns from Hybrid AI Practitioner-Responsible-Owners, generating commissions for genomic health predictive pre-eXam and eXam digital twin intercepts for health providers, while promoting public adoption of assured and explainable well-being, as depicted in Figure 1 and detailed in Tables 5–7.

Figure 3 Chart C, highlights innovative science and technology, transforming conventional primary care in a PHM mission for learning HPO as the national strategy (Putman et al., 2023; Department of Science, Innovation and Technology, 2023; United Nations, 2024; Genomics England, 2024a; GOV.UK and Industrial Strategy, 2025). Table 11 adoptions represent a strategic investment in an HPO digital twin, spanning from newborn screening to adult future health, with point-of-need predictors and intercepts in rare diseases and major conditions, supporting value-based care (Genomics England, 2024b; Our Future Health and NHS, 2022; Institute of Biomedical Sciences, 2023).

PHM integrates private-sector AI with public opinion on good health and well-being, using these as scientific data themes to evidence personalized features in the WGS Pre-eXam (UK Government, 2025). Scaling AI adoption for HPO ensures service adherence to better outcomes, whilst oversight on eXams intercepts requires national validation of the science data themes, AI architecture assurances, and the public right to choose (AI Safety Institute, 2024; UK Government, 2025). All the signals for primary care are the need for patient safety and implementation of HPO transformation through policy, as learning becomes evidence-based (Henry, 2025b; Henry, 2025c).

#### 5.3.1 Federated data platforms with human control

Government Principle 4 emphasizes the importance of having meaningful human control at the proper stages (Government Digital Service and Department for Science, Innovation and Technology, 2025). PHM Science and HPO Technology align the AIDRS on Federated Data Platforms to enable secure and safe health data sharing with meaningful human oversight in analysis across institutions (AI Safety Institute, 2024; NHSE, 2023a; Lefkowitz, 2019). The advantages of FDP in the context of PHM include meaningful human control, as detailed in Table 12.

**Table 12:** Federated data platforms provide meaningful human control for PHM.

Point	Adopt meaningful human control through FDPs for PHM
1	Data privacy and security using advanced encryption for WGS pre-eXam results
2	Enhance HPO-based BM accuracy, leading to precise care eXams
3	Mitigate bias by incorporating diverse datasets, ensuring fair AI for primary care
4	Scalable data processing, integrating large genomic datasets for analysis
5	Ensure compliance with data protection regulations and ethical standards
6	Collaboration among healthcare providers, improving HPO and PHM strategies
7	Efficient resource allocation, optimizing WGS pre-eXam and precise care eXams
8	Real-time data analysis for better decision-making based on HPO insights
9	Build public trust through transparency and enhance routine HPO/BM use
10	Supports HPO innovation and continuous improvement of primary care solutions

#### 5.3.2 Integrating genomic-phenotype health with AI solutions and skillsets

Government Principle 9 emphasizes the need to possess the skills and expertise required to implement and use AI solutions effectively through beginners, technical roles outside government digital analytics, data professionals, and leaders (Government Digital Service and Department for Science, Innovation and Technology, 2025). In Table 11 the PHM mission integrates genomic health with Gen AI, comprising teams with scientific and technical skills for BM. The rows in Table 11 outline the key technologies with the skillsets required under Principle 9 for the PHM of HPO with autonomous BM predictors and intercepts through agentic AI.

Finally, Table 11 identified several key solutions for sustainable good health, well-being, and economic growth. The author believes the three most important genomic-phenotype technological advances to realize classification capacity, capabilities, and privacy in the 10-year plan include the following:

- Quantum computing is revolutionizing BM with cutting-edge technology processes for complex health data, enabling more accurate intercepts and predictors (Deshprabhu, 2023). According to the National Quantum Computing Center, QC has shown significant potential in enhancing BM (Ur Rasool et al., 2023).
- GPT 4.5 is a series of LLMs developed by OpenAI that has significantly influenced healthcare’s ML and AI fields (Rabbani et al., 2025). GPT 5 represents the next iteration in the GPT series and is expected to be the best tool for HPO predictors and intercepts (Groza et al., 2024), as Gen AI [X] integrates pre-eXams/eXams task classifiers for adoption (Henry, 2025b).
- Federated learning enables the collaboration of multiple institutions to improve PHM while preserving the privacy and security of sensitive public citizen information, idealizing our robust digital twin (Dhade and Shirke, 2024). Federated Data Platforms offer a unique step-by-step process that engages stakeholders and public inclusiveness across all datasets in a secure PHM Mission with HPO policy (Stamatis et al., 2024; Henry, 2025c).

### 5.4 Continuous improvement and accountability

PHM development benefits from a U.K. independent report on AI opportunities in NHS adoption, shaping continuous improvement and accountability in technology finance, safe and trusted AI, and fit lifecycles (UK Government, 2025).

#### 5.4.1 Technology financial improvements and accountability

Figure 3 Chart B, highlights financial improvement and accountability by detailing the cost distribution across PHM development tasks, providing insights for achieving the Ten-Year Infrastructure Plan. Understanding the cost breakdown is essential for effective budget planning and resource management, ensuring long-term sustainability and maximizing investment impact (GOV.UK, 2023c; England, 2025b; Labour, 2023). Moreover, streamlining expensive processes, such as data acquisition and labeling, with standard open-source solutions such as PyTorch and TensorFlow aligned with national policy to reduce expenditure (Cardoso et al., 2022; Bansal, 2021; Henry, 2025c), as shown in Figure 2.

#### 5.4.2 Safe and trusted AI

Government Principle 5 outlines the entire AI lifecycle, including choosing the right tools, setting them up, maintaining, updating, and securely closing them down (Government Digital Service and Department for Science, Innovation and Technology, 2025). Table 1 defines a PHM mission to govern AI lifecycles for continuous HPO improvement, with safe and trusted AI delivered through Tables 2–4. Responsible owners are accountable for the predictors and intercepts, which rely on AI comparative analysis with continuous monitoring for bias mitigation (Pagano et al., 2022; Shah and Sureja, 2024; Vidyadhar Sribala et al., 2024; Birdi et al., 2024), promoting new surveys (Mehrabi et al., 2025), as shown in Tables 5–7 that continually drive efficiencies through HEMSS (Henry, 2025a).

Safe and Trusted AI is continually innovating, as shown in Table 11, requiring increased capacity to accelerate the PHM mission for federated HPO. Time is needed to develop and implement well-designed regulations and practical assurance tools. Regulators support innovation while ensuring safe and trusted AI development integrates personalized classification for continual improvement in the human lifecycle (UK Government, 2025). The government must protect citizens from significant AI risks and sustain public trust (Government Digital Service and Department for Science, Innovation and Technology, 2025). The regulation, safety, and assurance of classifiers in predictive WGS pre-eXams and valid digital twin eXam intercepts are ready for implementation to benefit the public with Agentic AI globally (Henry, 2025b; Henry, 2025c; Henry, 2025a).

HPO learning on evidence-based classifications, regulation, and support programs must ensure continuous AI improvement in key areas, such as BM, by presenting fit lifecycles for future analytics through open-source frameworks (Python, 2024; Taylor-Weiner et al., 2019; Cursa, 2024; Bansal, 2021; Cardoso et al., 2022), as illustrated in Figure 2. Sector regulators must be fit for the AI age by focusing on safe BM implementation through classification (Henry, 2025b). Collaboration with regulators such as AIDRS is needed to accelerate priority sectors through predictors and intercept initiatives in future regulatory sandboxes under HPO policy (UK Government, 2025; Henry, 2025c). Healthcare regulators, as suggested by Matt Clifford on behalf of the DHSC, must review initiatives such as HEMSS to address concerns and focus on HPO policy for value-based care at the point of need and to do that globally (UK Government, 2025; Institute of Biomedical Sciences, 2023; Henry, 2025a).

#### 5.4.3 Fit life cycles in analytics

Government Principle 5 emphasizes the importance of monitoring and managing drift, bias, and other anomalies to ensure accountability (Government Digital Service and Department for Science, Innovation and Technology, 2025). Gen AI assistants benefit from designs to perform and report repetitive WGS Pre-eXam tasks, reducing practitioners’ responsibility for genomics queries and improving health while lowering socioeconomic expense through pangenome specificity (Taylor et al., 2024). An AI diagnostics fund supports the deployment of national AI imaging technologies for faster diagnosis and treatment (Rong and Liu, 2024; NPIC, 2024). Access to biobanks and GP welfare records informs on social and environmental factors in a PHM mission (De Souza and Greenspan, 2025).

Accountable citizens adopt predictors and intercepts by embracing AI to improve public health life cycles with truth at the point of need (UK Government, 2025; Institute of Biomedical Sciences, 2023). By adopting AI-driven health assessments and interventions, citizens actively participate in their healthcare journey, ensuring responsible and ethical use of AI technologies (UK Government, 2025). Public trust in personalized technology is paramount, with citizens informed about AI’s benefits and potential HPO risks (UK Government, 2025). Government principles explain how Gen AI automatically enhances disease predictors to schedule intercepts with personalized therapies (Government Digital Service and Department for Science, Innovation and Technology, 2025). Responsible AI development and adoption is a joint responsibility of the digital workforce, health practitioners, and the public (UK Government, 2025; Government Digital Service and Department for Science, Innovation and Technology, 2025), while this transformation requires national policy and global stewardship (Henry, 2025b; Henry, 2025c; Henry, 2025a).

In Table 9, the author proposes an executive arm to the AIDRS/AISI that expedites regulations, standards, and tools on federated platforms to accelerate PHM mission adoption (The AI and Digital Regulations Service, n.d.; AI Safety Institute, 2024; NHSE, 2023a). Table 9 presents the HIMSS maturity, underpinning the Government’s Digital Regulation Service principles and the Independent Report adoption mission (UK Government, 2025; Government Digital Service and Department for Science, Innovation and Technology, 2025). At the same time, principles in HEMSS steward public inclusiveness, stakeholder engagement, and adherence to classification adoption, which accelerate the PHM mission from Genomic Newborn Screens (Henry, 2025). AIDRS/AISI Agentic AI analytics steward PHM with HEMSS, which cultivates HPO workflow in a life cycle of continuous improvement (The AI and Digital Regulations Service, n.d.; AI Safety Institute, 2024; NHSE, 2023a; Henry, 2025b; Henry, 2025c; Henry, 2025a; Henry, 2023).

## 6 Conclusion

Sections 2, 3 aim to integrate scientific data into engineered infrastructure for agile PHM, thereby enhancing public health and patient safety through AI technologies that steward efficient HPO as primary care, ensuring truth at the point of need. Personalized aims achieved through AI-driven interventions focus on predictors to individualize intercepts as value-based care. Ensuring high-quality data training in the ecosystem and providing accurate and reliable Pre-eXam and eXams is crucial for assurance. HPO analytic projects are stewarded to ensure PHM’s safety and ethical deployment, thereby improving health outcomes with public trust in a secure environment.

Section 4, 5 emphasizes new governance, continuous improvement, and accountability in implementing fit lifecycles in analytics. It outlines the need for a robust infrastructure, strategic alignment, and ethical oversight to ensure the successful integration of AI into PHM. By aligning with government AI principles and focusing on federated data platforms and emerging technologies, the section highlights the potential for transforming PHM through advanced analytics and personalized care. Training, engaging stakeholders, and aligning HPO policies with technological advancements are underscored to achieve long-term sustainability and resilience in healthcare.

The manuscript highlights the alignment of science and technology within PHM, effectively classifying and deploying reforms progressively in a five-point plan that expands in the digital space. As the fifth article in a series of six, it explores the transformative potential of integrating genomic health Pre-eXams and precise care eXams into PHM. This HEMSS program is dedicated to cultivating, transforming, and improving newborn screening and adult health journeys in an era of healthcare reform. The strategic alignment of AI and PHM ensures innovative, equitable, and inclusive healthcare delivery.

The PHM transformative potential of GPT-5, security of Federated Learning, and Quantum Computing with Whole Genome Sequencing (WGS) in HPO is explored. GPT-5’s advanced language processing analyzes vast medical literature and clinical data to personalize health. Federated learning ensures data privacy by training AI models without sharing sensitive information. QC rapidly solves complex computations, accelerating pattern recognition in genomic data analysis and HPO computations. These technologies create a powerful synergy, revolutionizing PHM by providing personalized, data-driven insights, enabling early disease detection and prevention, and ultimately enhancing human well-being.

The conclusion emphasizes the potential of the proposed PHM strategy to align the UK AI Action Plan as a national mission for scientific research integration with technical principles outlined in the UK Government AI Playbook. Integrating scientific data themes into evidence-based primary care practice accelerates agile capabilities, advancing public health and patient safety for national well-being and growth. As technology capacity increases, value-based care will be demonstrated through genomic medical science and HPO with expert AI capabilities in Pre-eXam and eXam classifications.

## Glossary

AIDRS: UK AI Digital Regulation Service
AISI: AI Safety/Security Institute
AI: Artificial Intelligence
BM: Biological Modeling
CCM: Continuous Control Monitoring
CNN: Convolutional Neural Network
DL: Deep Learning
DSIT: Department for Science, Innovation and Technology
FDP: Federated Data Platforms
GANs: Generative Adversarial Networks
Gen AI: Generative AI
HEMSS: Higher Expert Medical Science Safety
HIMSS: Healthcare Information and Management Systems Society
HPO: Human Phenotype Ontology
HRA: Health Research Authority
ICB: Integrated Care Board
NICE: National Institute for Health and Care Excellence
NIHR: National Institute for Health and Care Research
NIST: National Institute of Standards and Technology
NLP: Natural Language Processing
PHM: Population Health Management
QA: Quality Assurance
SHAP: SHapley Additive exPlanations
SM: Structural Monitoring
ViTs: Visual transformers
WGS: Whole Genome Sequencing
XAI: Explainable AI
